# Differences in Nutritional Composition of *Poria cocos* Cultivated with Different Raw Materials Based on Non-Targeted Metabolomics Method

**DOI:** 10.3390/jof12090637

**Published:** 2026-08-26

**Authors:** Yusong Li, Jianbin Xu, Chunlai Yu, Jinping Zhang, Yinan Wang, Zeyu Zhang, Fengqing Li, Kaitai Yang

**Affiliations:** 1 Subtropical Forestry Research Institute, Chinese Academy of Forestry, Hangzhou 311400, China; yusongli2004@163.com (Y.L.);; 2 College of Landscape Architecture, Nanjing Forestry University, Nanjing 210037, China; 3 Chun’an County Forestry Bureau, Hangzhou 311700, China; 4 Experimental Center of Subtropical Forestry, Chinese Academy of Forestry, Xinyu 336603, China; 5 Guanxi Zhuang Autonomous Region Academy of Forestry, Nanning 530002, China

**Keywords:** *Poria cocos*, pine wilt woods, bag material cultivation, non-targeted metabolomics analysis, N/P ratio

## Abstract

The spread of *Bursaphelenchus xylophilus* has caused a critical shortage of traditional *Poria cocos* cultivation materials, making bag-based substrates an urgent alternative. Yet, how substrate stoichiometry shapes nutritional quality remains unclear. Using non-targeted metabolomics combined with redundancy analysis (RDA) and weighted gene co-expression network analysis (WGCNA), we profiled *P. cocos* cultivated on four substrates: healthy pine logs, pine wilt wood bags, oak bags, and pine needle/branch bags. Bag-cultivated *P. cocos* showed significantly elevated total amino acids, poria cocos acid, and total triterpenoids, with pine wilt wood bags (P1) performing best overall. Nitrogen, phosphorus, and the N/P ratio independently drove metabolomic variation (pairwise overlap < 5%). Nitrogen-line hub metabolites were negatively correlated with amino acid content, suggesting that suppressed lipid metabolism may free carbon skeletons for the accumulation of nitrogenous nutrients. Phosphorus-line hub metabolites were positively associated with polysaccharide indices, whereas the N/P ratio in line lipid amides showed strong negative correlations with polysaccharides under phosphorus limitation. These correlational patterns are consistent with a stoichiometric resource allocation model, although direct validation through controlled-element experiments is required. These findings provide quantitative guidance for optimizing bag-substrate formulations in *P. cocos* cultivation.

## 1. Introduction

*Poria cocos* is a type of porous fungus that can be used as medicine and food. In the natural environment, *P. cocos* is usually parasitic on *Pinus massoniana* trees and distributed across China, North Korea, Japan, and North America [1]. This precious fungus is rich in chemical components, including a large number of terpenoids and polysaccharides [2]. It has an abundant amount of amino acids, proteins, poria cocos acid, and other substances [3]. Therefore, these components provide *P. cocos* with a variety of beneficial biological activities, including anti-tumor, anti-inflammatory, immunomodulation [4,5,6], and antioxidant properties, as well as the ability to relieve blood pressure [7]. In addition, polysaccharides from *P. cocos* are an excellent raw material for antibacterial films used for food [8]. Because of its excellent medicinal and commercial value, *P. cocos* has huge demand in domestic and foreign markets.

*P. cocos* is an important fungus both in medicine and food, and its quality formation is closely related to the cultivation materials. Traditionally, *P. cocos* cultivation mainly depends on healthy *Pinus massoniana* stems, supplemented by healthy pine roots [9]. In recent years, *Bursaphelenchus xylophilus* disease has spread widely; as a nematode, it decreases plant hormone synthesis and ultimately affects pine growth [10]. The infected pine trees continue to impact the environment because they carry a large number of longicorn larvae and *Bursaphelenchus xylophilus* eggs [11], which cannot be used in the cultivation of *P. cocos*, resulting in a serious shortage of raw materials for *P. cocos* cultivation. In order to solve this dilemma, researchers explored the feasibility of using pine residues (such as pine segments, pine wilt wood, pine needles, pine branches, etc.) and other tree species (hardwood such as oak) to formulate bags for the cultivation of *P. cocos*. Bag-based cultivation differs significantly from log-based cultivation due to the addition of auxiliary materials to crushed wood raw materials to support mycelium growth [9]. This results in notable variations in chemical composition and nutritional content. For example, the inclusion of rice bran and corn kernels in bag-substrate varieties enhances the levels of vitamins and minerals available and regulates the C/N ratio. Moreover, in bag-substrate varieties, pH was increased by incorporating gypsum, thereby enhancing its buffering capacity [9].

Different cultivation substrates can significantly influence the yield and endogenous metabolites of *P. cocos*. After adding straw and rice bran to the cultivation substrate, the output and quality of *oyster mushroom* significantly improved [12]. These differences in the cultivation substrates lead to differences in the efficacy of *P. cocos*. Additionally, studies utilizing animal models demonstrated that an extract of *P. cocos*, cultivated by traditional logs and bag materials, can notably enhance the xenogeneic resistance of mice with compromised immune function while reducing levels of interleukin-2 and tumor necrosis factor α [13]. However, existing studies mostly focus on regional differences [14] and effective components and medicinal mechanisms [5,7]. Research on the relationship between cultivation raw materials and metabolic characteristics is insufficient. Consequently, there is a notable lack of comprehensive analyses that evaluate the full spectrum of endogenous metabolites.

Metabolomics is a new research method developed after genomics, transcriptomics, and proteomics. It has been widely used in various fields, from nutrition and health to the design and optimization of cell factories [15]. At present, high-performance liquid chromatography (HPLC) or gas chromatography (GC) combined with high-resolution mass spectrometry (MS) is widely used to identify small-molecular metabolites with molecular weights less than 1500Da in various organisms. At the same time, metabolites are screened and identified using multivariate statistical analysis. Liu et al. used UHPLC–ESI–Q–Orbitrap–MS and metabolomics analysis to screen out potential biomarkers that could be traced back to the production area of *P. cocos* [14]. Schueuermann et al. used gas chromatography–mass spectrometry (GC-MS) combined with metabolomic data analysis to identify potential volatile fungal marker compounds in the juice of Chardonnay grapes infected by different fungi [16]. This shows that chromatography–tandem mass spectrometry combined with metabolomics is widely used and reliable in screening representative metabolites. However, the research on representative metabolites of *P. cocos* cultivated with different raw materials has not yet been reported.

In this study, pine wilt wood, pine needles, and oak were used as the main raw materials, supplemented by corn kernels and rice bran, and bag materials were prepared to cultivate *P. cocos*. The stoichiometry of the cultivated raw materials, along with the nutritional components and endogenous metabolites of *P. cocos* grown in bag materials and those cultivated on healthy pine trees, were qualitatively analyzed. Non-targeted metabolomics was employed to examine the differences in *P. cocos* metabolites cultivated with various raw materials. This study aims to identify the reasons behind these different nutritional components in the four groups. This strategy provides an optimized approach for studying *P. cocos* formulas in bag culture.

## 2. Materials and Methods

### 2.1. Cultivation and Sampling

The test site is located in Yuexi County, Anhui Province (32°12′ north latitude and 116°23′ east longitude, with an altitude of 550 m), with an average temperature of 22–28 °C and relative humidity of 65–85% during the cultivation period (May–November 2023). The cultivation cellars were placed under pine forest with 60% light reduction. *P. cocos* strain CGMCC5.78 (China General Microbiological Culture Collection Center, CGMCC) was inoculated into four different cultivation materials: traditional cultivation using healthy pine segments as cultivation materials (CK); bag material using pine wilt wood as the cultivation material (P1); bag material using oak trees as the cultivation material (P2); and bag material using pine branches and needles as the cultivation materials (P3). The cultivation methods are as follows: The CK group consists of healthy *Pinus massoniana* stems with a diameter of 25 cm, cut to 45 cm, and weighed in 6 kg pine segments for one cellar. They are inoculated with *P. cocos* strain CGMCC 5.78 in the top side of pine logs straightly, covered with soil for 5–8 cm, grafted with 50 g fresh *P. cocos* sclerotia (cultivated from *P. cocos* CGMCC 5.78) for each cellar on the bottom side of pine logs 15 days later. The formula of bag material followed that of Yinan Wang [9]: 76% wood chips, 12% rice bran, 10% corn kernels, 1% sucrose, and 1% gypsum. Trees were pulverized into 10 mm chips before mixing with other materials according to the formula; the water content was adjusted to 60%, substrates were placed into fungal bags (bag size is 20 × 60 cm), and the sample was sterilized at 100 °C for 12 h. Then, inoculation of the *P. cocos* strain was performed when the bag cooled to room temperature, then, cultivated in culture room (25 °C, humidity 60%) for 15 days. In total, 6 kg of bag material was weighed for one cellar; these were grafted with 50 g of fresh *P. cocos* sclerotia for each cellar, covered with soil for 5–8 cm, and harvested after 6 months (the sclerotia epidermis is yellowish-brown with no white cracks). Each cultivation group comprised 30 independent cultivation cellars. Six different *P. cocos* were selected randomly from each group after harvest; the skin of *P. cocos* was peeled, and the sclerotia (white *Poria*) were ground into powder (each composite sample was independently extracted and analyzed), and then placed at −4 °C for later use.

### 2.2. Stoichiometric Analysis and Nutrition Analysis

The C content of the pine and bag material was determined using the potassium dichromate volumetric external heating method. The contents of P and N were determined using the sulfuric acid–hydrogen peroxide digestion method and semi-micro Kjeldahl nitrogen determination method, respectively [17].

The moisture and alcohol-soluble contents of *P. cocos* were determined using the hot soaking method according to the general principles of the Chinese Pharmacopoeia [18]. The content of ash was determined by high-temperature incineration at 550 °C according to Chinese national standards (GB 50009.4-2016) [19]. Total polysaccharide was determined by the Fehling’s reagent method (GB/T 15672-2009) [20]. Total amino acids determined by post-column ninhydrin derivatization ion-exchange chromatography following acid hydrolysis (6 M HCl, 110 °C, 22 h; GB/T 5009.124-2016) [21]. The contents of poria cocos acid and total triterpenes were determined using HPLC and ultraviolet (UV) spectrophotometry, respectively [9].

### 2.3. Preparation of Samples for Metabolomics Analysis

A 100 mg aliquot of sample powder was weighed into a 10 mL centrifuge tube, followed by the addition of a 5 mL extraction solvent (methanol–water, 3:1, *v*/*v*). The mixture was homogenized and kept at 4 °C overnight under continuous stirring. After that, 1.5 mL of the supernatant was transferred into a new centrifuge tube and centrifuged (4 °C, 10,000 rpm) for 10 min. The resulting supernatant was filtered through a disposable 0.22 μm cellulose acetate membrane and transferred into a 2 mL HPLC vial. To ensure data quality in metabolomic profiling, a quality control (QC) sample was prepared by combining equal aliquots from all analytical samples; this was employed for instrument performance monitoring and data normalization. The QC sample was analyzed once every six experimental injections, along with a solvent blank processed in parallel. All prepared samples were analyzed using a UHPLC–ESI–Q–Orbitrap–MS system. Three technical replicates were performed for each sample to ensure reproducibility.

### 2.4. UHPLC–ESI–Q–Orbitrap–MS Analysis

Throughout the UHPLC–ESI–Q–Orbitrap–MS analysis, the samples were placed in an automatic sampler at 4 °C, and the metabolic components were analyzed using the UltiMateTM 3000 ultra-high-performance liquid system (Thermo Scientific, San Jose, CA, USA) and Q-Exactive Plus (Thermo Scientific, San Jose, CA, USA). Chromatographic separation was achieved on a Waters Acquity HSS T3 column (2.1 mm × 150 mm, 1.8 µm, Waters Technology Co. Ltd., Milford, NH, USA) at a flow rate of 0.3 mL/min. After equilibration at 25 °C, each sample was injected in a 3 μL volume. For the mobile phase, phase A is 0.1% formic acid aqueous solution, and phase B is 0.1% formic acid acetonitrile solution. The chromatographic conditions of gradient elution are 2% B for 3 min; an increase to 98% B within 12 min, maintained for 2 min; and then a sharp decrease in B from 98% B to 2% in 0.5 min. Finally, it was stabilized at a volume ratio of 2% B for 2.5 min, and the total operation time of each sample was 20 min.

Mass spectrometric detection was conducted in both positive and negative ionization modes using electrospray ionization (ESI). The capillary voltage was set to 3.5 kV for both modes. The ion transfer tube temperature was maintained at 320 °C, and the auxiliary gas heater temperature was set to 400 °C. Sheath gas and auxiliary gas flow rates were set at 40 and 10 (arbitrary units), respectively. The full MS/dd-MS^2^ (Top N) scanning mode was applied with a mass range of *m*/*z* 60–900. The AGC target was set to 1 × 10^6^ for full MS and 5 × 10^5^ for dd-MS^2^, and the maximum injection time was set to automatic. The precursor isolation window for dd-MS^2^ was 1.0 *m*/*z*. The resolution was set to 70,000 for full MS and 15,000 for MS/MS. Normalized collision energies (NCEs) of 20, 40, and 60 were used for stepped fragmentation.

### 2.5. Qualitative and Quantitative Analysis of Metabolites

Xcalibur2.1.1 software (Thermo Fisher Company, Newark, DE, USA) was used to collect the data of the original spectrogram, and CompoundDiscoverer3.2 software (Thermo Fisher Company, Waltham, MA, USA) was used to process the original data. The solvent blank sample was used to eliminate background noise interference, and the QC sample was used in the whole process of sample operation to ensure the validity and stability of the test data. Finally, the compounds covered by more than 50% of QC samples and with a relative standard deviation (RSD) less than 30% were screened. Screening conditions included accurate molecular weight deviation (≤5 ppm), retention time correction (≤0.2 min), peak response intensity (≥200,000), background signal-to-noise ratio (SN > 3), and so on. Compound identification followed Metabolomics Standard Initiative (MSI) guidelines at three confidence levels: Level 1: matched to in-house reference standards by retention time, accurate mass (Δ < 5 ppm), and MS/MS spectra; Level 2: putatively annotated by spectral library matching against mzCloud (https://www.mzcloud.org, accessed on 1 August 2025), METLIN (https://metlin.scripps.edu, accessed on 1 August 2025), KEGG (https://www.genome.jp/kegg, accessed on 1 August 2025) and HMDB 5.0 (MS/MS similarity ≥ 70%); Level 3: putatively characterized by accurate mass and isotope pattern matching to the above databases.

### 2.6. Data Analysis

A machine imported the data into an Excel table and used the peak area as the relative content index of metabolites. The data matrix was uploaded to MetaboAnalyst 6.0 for systematic clustering analysis (HCA) and KEGG pathway enrichment analysis. TBtools-II (version 2.303) was used to draw the Venn diagram and cluster heat map, and Origin Pro 2021 software (Origin Lab Corp, Northampton, MA, USA) was used to draw the box diagram and bubble diagram. The Mantel test and WGCNA were performed using the Metware Cloud, an online platform for data analysis (https://cloud.metware.cn, accessed on 15 August 2025 ). Redundancy analysis (RDA) was performed using R (version 4.5.1).

## 3. Results

### 3.1. Stoichiometric Analysis of Raw Materials

**Figure 1:**
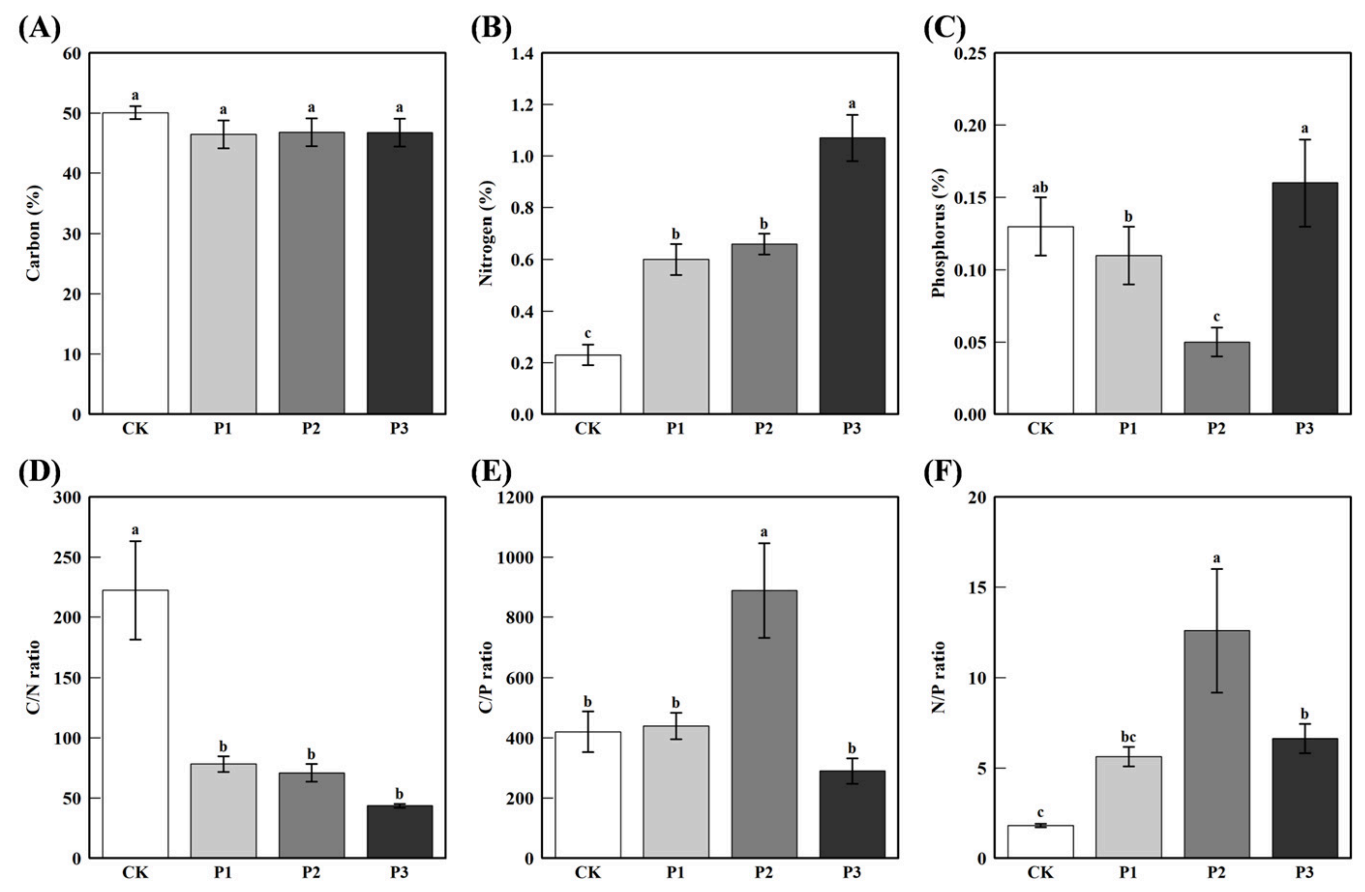
Chemometric characteristics of CK (traditional cultivation using healthy pine segments as cultivation materials), P1 (bag material using pine wilt wood as the cultivation material), P2 (bag material using oak trees as the cultivation material), and P3 (bag material using pine branches and needles as the cultivation materials): (**A**) carbon content; (**B**) nitrogen content; (**C**) phosphorus content; (**D**) C/N ratio; (**E**) C/P ratio; (**F**) N/P ratio. Data are means of 3 replicates ± SE. Different lowercase letters indicate significant differences (Duncan, *p* < 0.05) among different samples.

It was found that the elements C, N, P, and their ratios in the growth substrate affected the growth and composition of microorganisms [22]. The stoichiometric characteristics (contents of carbon, nitrogen, phosphorus, and their ratios) in the cultivation materials of *P. cocos* are shown in Figure 1. The carbon content in the bag material and CK was similar. The nitrogen in the bag material was significantly higher than that in CK, with the highest content noted in the P3 group (1.07%) and the lowest noted in the P1 group (0.60%). The phosphorus contents in the bag material fluctuated less than those in the wood segment group. It is worth noting that the phosphorus in P2 (0.05%) is significantly lower than that in the other groups. The significant difference in nitrogen and phosphorus between the bag materials and CK resulted from the inclusion of corn kernels and rice bran in the bag materials. Furthermore, the nitrogen and phosphorus contents in three bag materials differed due to variations in the components of pine wood at different parts and between pine and oak wood. The ratio of nitrogen to phosphorus (N/P) in the bag material group was significantly higher than that in the CK group, with the highest ratio being in the P2 group (12.59) and the lowest ratio being in the P1 group (5.63).

### 3.2. Nutrition Composition of P. cocos

Compared with *P. cocos* planted directly in wood segments, *P. cocos* cultivated with bag materials has a certain increase in many nutrients (Table 1). The contents of total triterpenoids, poria cocos acid and total amino acids in bag material were significantly higher than those in CK, with the highest content of total triterpenes being in the P2 group (0.8284%), the highest content of poria cocos acid (783.94 mg/kg) and the highest biological conversation efficiency (31.72%) being in the P1 group, and the highest content of total amino acids being in the P3 group (7.58%). However, water-soluble polysaccharide (WPCP) and alkali-soluble polysaccharide (APCP) in *P. cocos* cultivated with bag materials were significantly lower than those in CK, with the lowest WPCP (0.3568%) and APCP (39.27%) in the P2 group. Alcohol-soluble substances significantly decreased in the P2 group (1.70%) compared to CK, which did not meet the minimum standard (2.50%) of the People’s Republic of China (PRC) Pharmacopoeia [18]. This result indicates that compared with CK, bag-based cultivation with pine wilt wood, pine needles, and pine branches generated a slightly lower number of polysaccharides but a significant increase in poria cocos acid, triterpenoids, and total amino acids, as well as biological conversation efficiency. Among the bag material groups, P2 produced *P. cocos* with the lowest quality in terms of alcohol-soluble substances, poria cocos acid, WPCP, and APCP. However, the triterpenoids, poria cocos acid and total amino acids of *P. cocos* in the P2 group were higher than those in the pine log group, making this *P. cocos* of high edible nutritional value.

### 3.3. Metabolic Composition of P. cocos

#### 3.3.1. Hierarchical Cluster Analysis of Endogenous Metabolites

UHPLC–ESI–Q–Orbitrap–MS was used to explore the differences in endogenous metabolites of *P. cocos* cultivated with four cultivation materials. The maximum response values for the four groups in ESI+ and ESI- modes were all above 3 × 10^9^. This implies that the detection signal intensity meets the requirements of routine analysis and quality supervision. The signal intensities in positive- and negative-ion modes were similar, indicating that most compounds present response values in both modes. Mass spectrometry identified 4705 positive-ion characteristic peaks and 4283 negative-ion characteristic peaks, with 576 endogenous metabolites under positive and negative ions.

**Figure 2:**
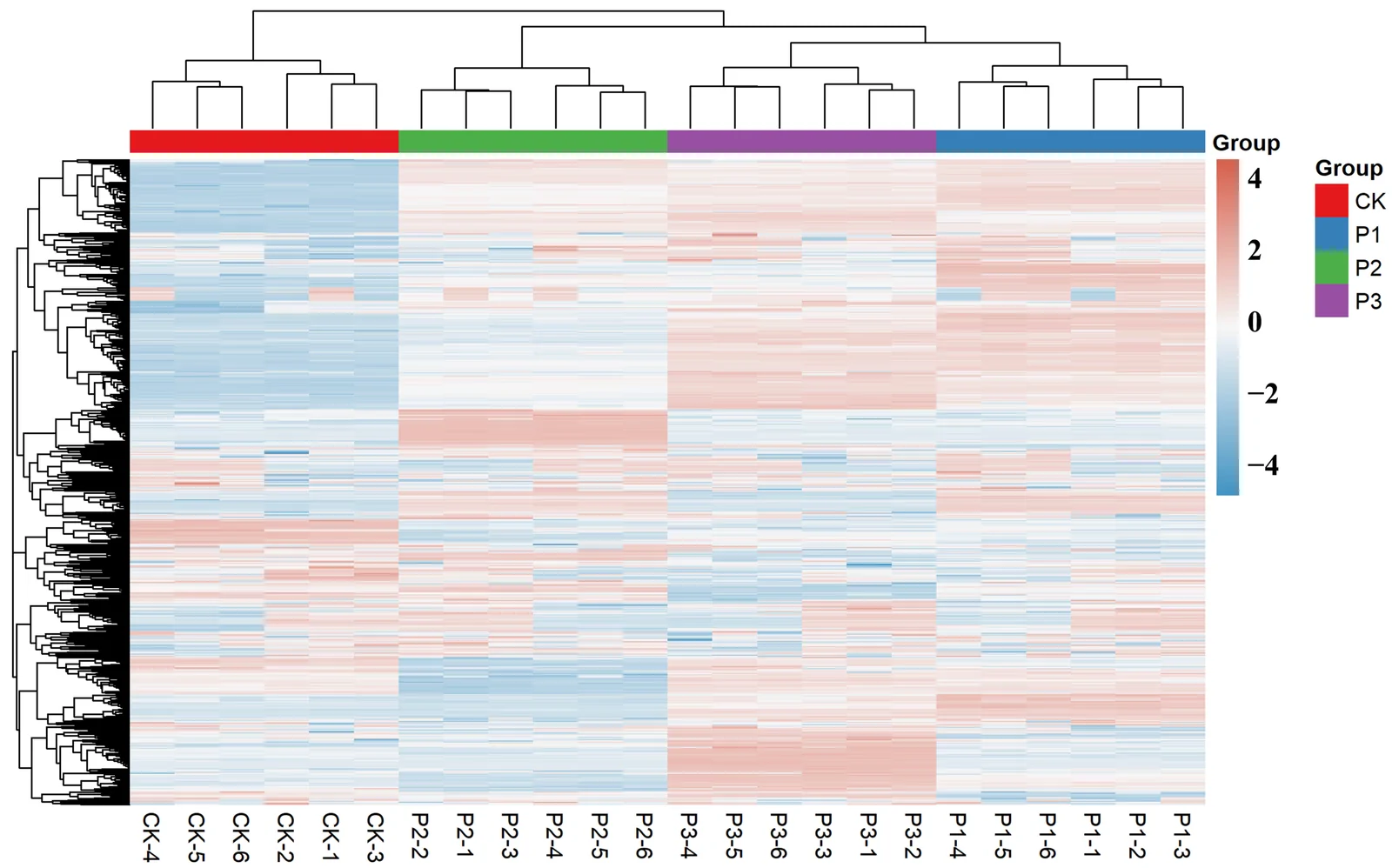
Hierarchical cluster heatmap: CK (traditional cultivation using healthy pine segments as cultivation materials, red), P1 (bag material using pine wilt wood as the cultivation material, blue), P2 (bag material using oak trees as the cultivation material, green) and P3 (bag material using pine branches and needles as the cultivation materials, purple) were clustered in this heatmap using red and blue; the red color shows a higher metabolite expression level, and the blue color shows a lower metabolite expression level.

Based on differences in metabolite expression levels, four groups were classified and clustered, with the results visualized using a hierarchically clustered heatmap (Figure 2). Metabolites in the same cluster indicate that they undergo similar reaction steps in the metabolic pathway. Based on the differences in endogenous metabolites, the four groups of *P. cocos* samples were divided into two categories: the CK group as one category and P1, P2, and P3 clustered into another category. P1 and P3 were clustered in the same category, with P2 in a separate category. This indicates that the expression of *P. cocos* endogenous metabolites in the CK and bag material groups varied. The metabolic level in CK was generally low, while most endogenous metabolites in P1, P2, and P3 were relatively high. P2 had a different metabolic module than P1 and P3.

#### 3.3.2. RDA and Variance Decomposition

**Figure 3:**
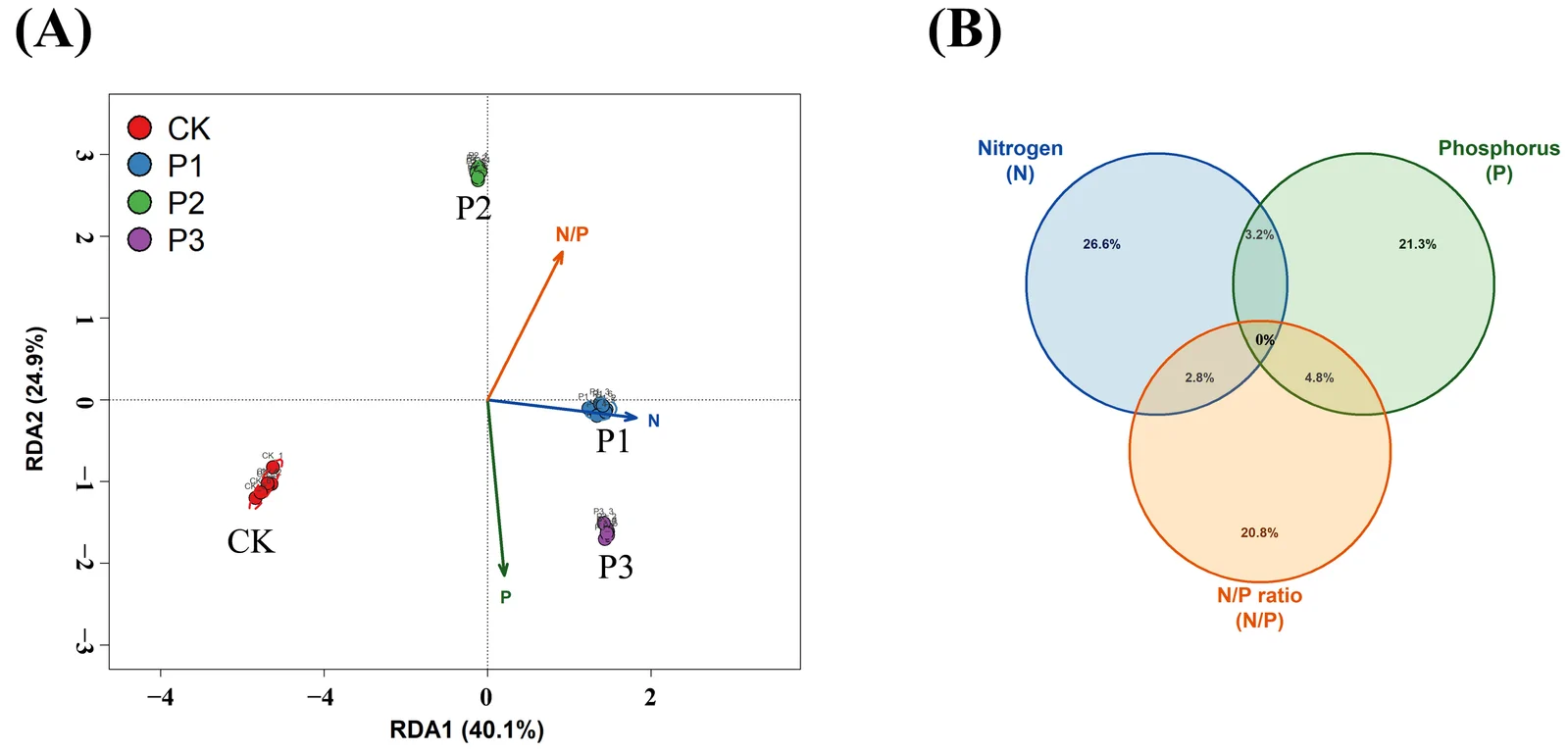
RDA. (**A**) RDA biplot constrained by N, P, and the N/P ratio (F = 22.34, *p* = 0.001, R^2^adj = 0.736): Vectors indicate the direction and strength (length) of each stoichiometric factor; colors correspond to N (blue), P (green), and the N/P ratio (orange). (**B**) Variance partitioning of the metabolome by N, P, and N/P ratio.

The RDA chart reflects the influence of the physical and chemical properties of the substrate on the overall metabolites of *P. cocos*. Carbon content did not differ significantly among substrates (*p* > 0.05, Table 1) and was therefore excluded from the RDA model to avoid spurious variance attribution. As shown in Figure 3A, the full RDA model is highly significant (F = 22.34, *p* = 0.001, 999 permutations), explaining 73.6% of metabolomic variance (R^2^adj). The N element points in the positive direction of RDA1 (40.1%), indicating that its inclusion in the matrix is the main positive correlation factor causing the P1 and P3 groups to diverge from CK. The P element points in the negative direction of RDA2 (24.9%), indicating that this is the main negative correlation factor that causes P2 to diverge from CK, and the main reason for the separation between the P1 and P3 groups. Based on the RDA model, a variance decomposition calculation (VPA) was performed on the data. The results are shown in the Venn diagram in Figure 3B. Among them, N (26.6%), P (21.3%), and N/P (20.8%) have different independent contribution rates to the RDA model, and the overlap is extremely low (0%), indicating that the three are not in the matrix.

#### 3.3.3. WGCNA

**Figure 4:**
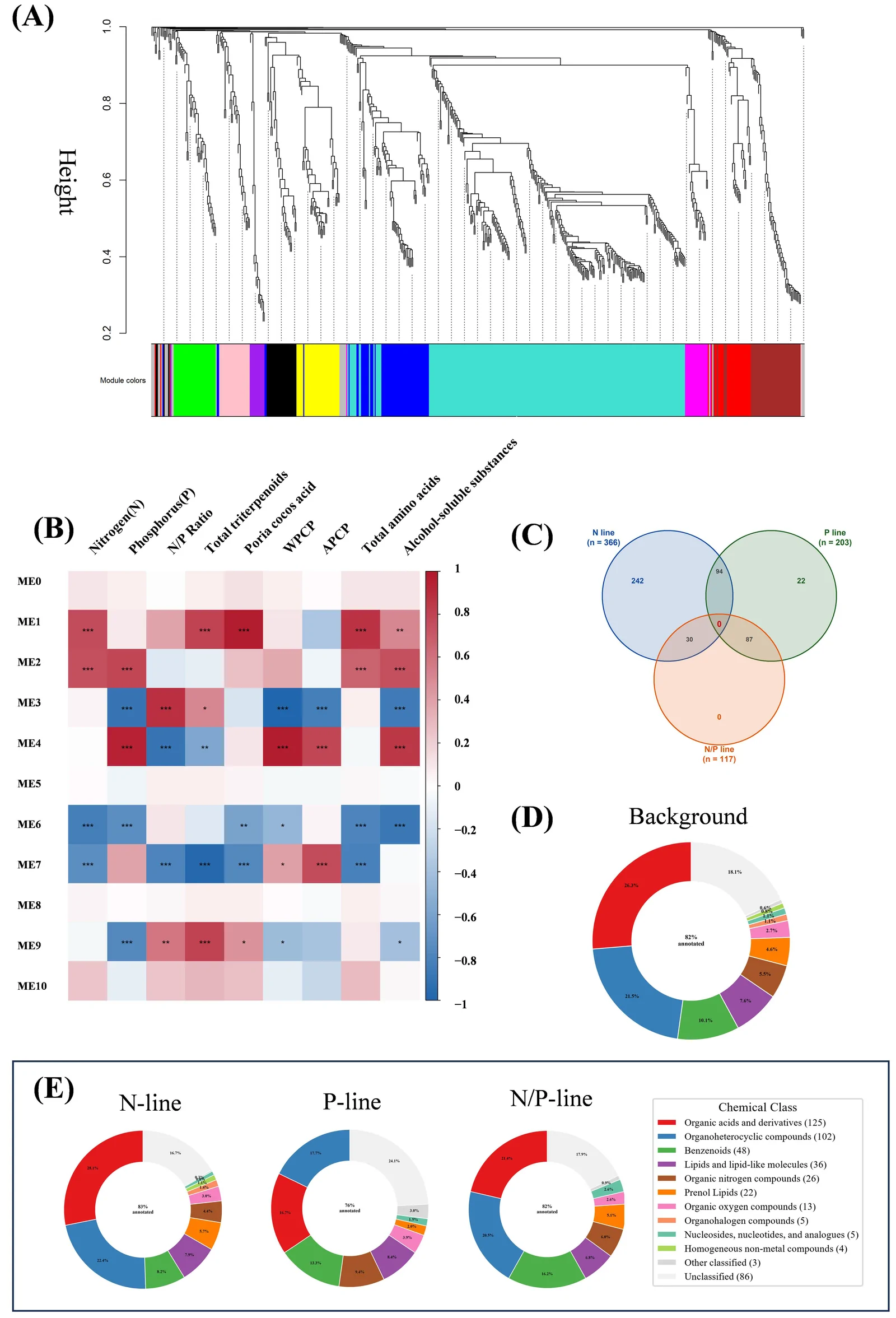
WGCNA: (**A**) Clustering dendrogram of metabolites, different colors represent different metabolite modules (ME); (**B**) module–trait correlation heatmap, In the heatmap, red indicates positive correlation and blue indicates negative correlation. Asterisks indicate significant differences (* *p* < 0.05, ** *p* < 0.01, *** *p* < 0.001); (**C**) three-line Venn diagram showing the overlap of metabolites assigned to N line, P line and N/P ratio lines. (**D**,**E**) Pie diagram: Chemical class distribution of metabolites in the background, N line, P line and N/P ratio line.

WGCNA is often used for gene co-expression network analysis. This paper uses WGCNA to construct a metabolite co-expression clustering dendrogram (Figure 4A) and identifies 11 metabolite modules (ME0–ME10) with similar expression patterns. A total of four modules (ME0, ME5, ME8, and ME10) had no significant correlation with the chemical elements in the matrix and the chemical composition of *P. cocos* and were not involved in subsequent analysis. The module–trait correlation analysis heat map (Figure 4B) reveals the correlation between chemical elements and metabolite modules, among which N element response modules (ME1, ME2, ME6, and ME7; |r| > 0.7) are positively correlated with the accumulation of triterpenes and amino acids (r = 0.81–0.97), P element response modules (ME2, ME3, and ME4; |r| > 0.7) are significantly correlated with the accumulation of polysaccharide components in *P. cocos*, and the N/P ratio shows a relationship between chemical balance in the matrix. Here, modules ME3 and ME4 are shared with P elements in a completely opposite relationship, indicating that the N/P ratio affects the synthesis of polysaccharides mainly due to the limitation of P elements rather than N elements.

All metabolites in the module are classified into stoichiometric lines based on the correlation coefficient between different modules and chemical elements (|r| > 0.7). There are 366 metabolite responses in the N element line, 203 metabolite responses in the P element line, and 117 metabolite responses in the N/P ratio line, with no metabolite common to all three lines simultaneously. As shown in the Venn diagram (Figure 4C), the N and P lines only intersect with 94 metabolites, and the number of metabolites that the three lines intersect with is 0. This is consistent with the previous RDA results. The N and P lines regulate different metabolic pathways through various metabolite modules, respectively; the P line intersects with the N/P ratio line for 87 metabolites, and the N line intersects with the N/P ratio line for 30 metabolites. This shows that the impact of the N/P ratio’s chemical balance on the metabolites of *P. cocos* is mainly reflected in limited P elements.

#### 3.3.4. Hub Metabolite Classification

The compounds were annotated and interpreted using the three lines of N, P, and the N/P ratio. A total of 475 compounds were identified (Figure 4D,E). Due to the limitations of the database, only 81.9% (389) of the compounds were annotated by superclass, with a total of 11 superclass classifications: Organic acids and derivatives (26.3%), Organo heterocyclic compounds (21.5%), Benzenoids (10.1%), lipids and lipid-like molecules (7.6%), Organic nitrogen compounds (7.6%), and Prenol Lipids (4.6%) account for the main compound categories. In the N line, the proportion of acids and derivatives (28.1%) and Prenol Lipids (5.7%) is higher than the distribution trend of the overall compounds, which shows that under sufficient matrix N conditions, the synthesis of amino acids and terpenoid precursors of *P. cocos* is promoted. In the P line, Benzenoids (13.3%) and Organic nitrogen compounds (9.4%) increased, and Prenol Lipids (2.6%) decreased, which indicated that P affected the metabolism of N and inhibited the synthesis of terpenoid components in *P. cocos*. In the N/P ratio line, the proportion of Benzenoids (16.2%) increased significantly, indicating that the chemical dose coefficient ratio affects the metabolism of aromatic compounds. Further enrichment of the KEGG pathway for metabolites found that only two pathways were enriched in the N/P ratio pathway (*p* < 0.05). Steroid hormone synthesis is interconverted with pentose and glucuronic acid, which is related to the triterpene skeleton synthesis of *P. cocos* and the core pathway of polysaccharide metabolism.

#### 3.3.5. Hub Metabolite–Nutrient Correlation Analysis

**Figure 5:**
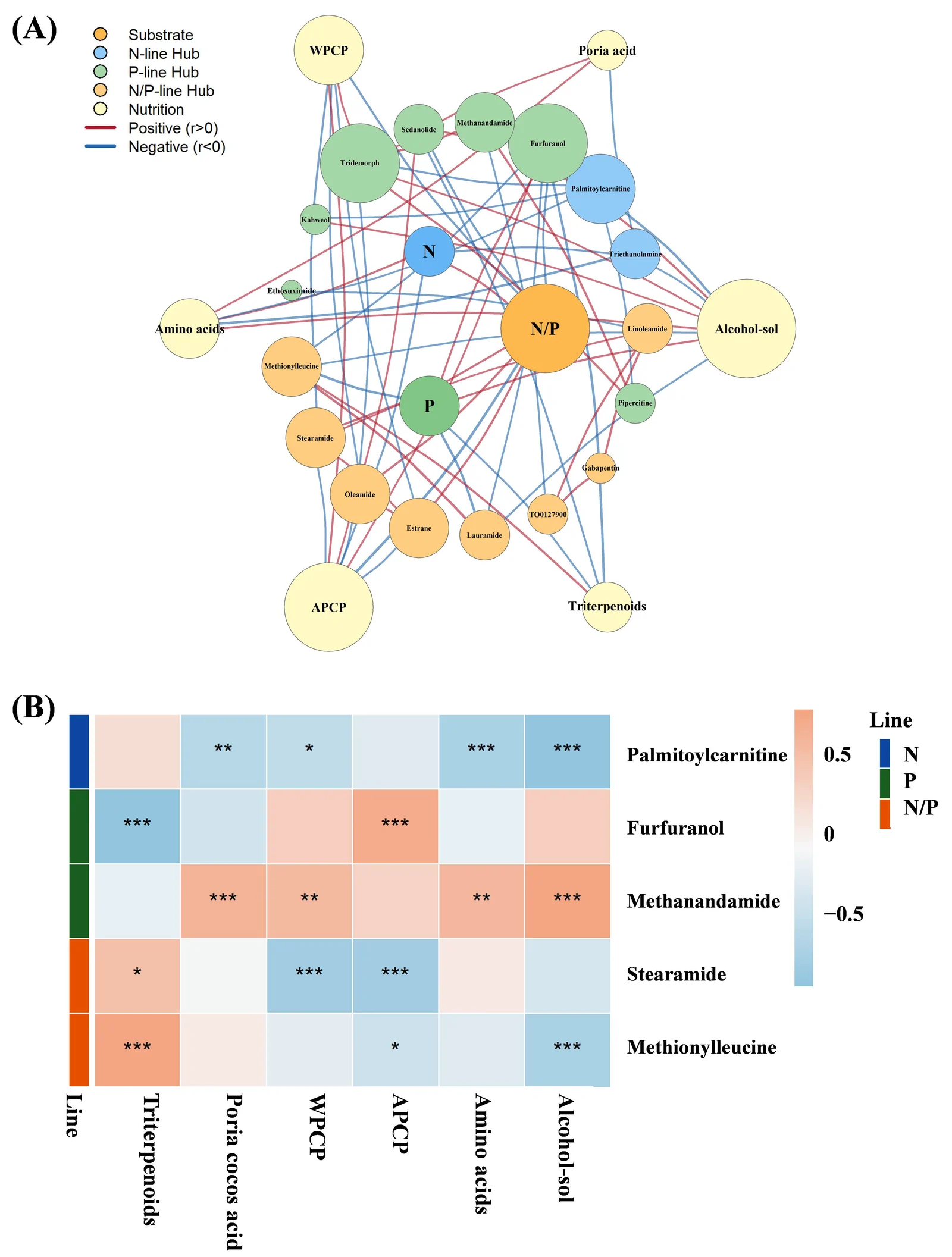
Correlation analysis. (**A**) Three-way association network: Integrating substrate factors (inner ring), hub metabolites (middle ring, *n* = 18), and nutritional quality traits (outer ring). Edges represent significant Spearman correlations (|r| > 0.7, *p* < 0.05); red edges denote positive correlations; and blue edges denote negative correlations. Node size is proportional to degree (number of significant edges) and edge width to |r|. (**B**) Representative hub metabolites: Spearman correlation heatmap between five representative hub metabolites and six nutritional quality traits. Hub metabolites were selected as those with |kME| > 0.8 within their respective WGCNA modules and belong to explainable chemical classes. Asterisks denote significance. Row colors indicate stoichiometric line assignment (blue, N; green, P; orange, N/P). Asterisks indicate significant differences (* *p* < 0.05, ** *p* < 0.01, *** *p* < 0.001).

In WGCNA, module membership (MM, kME) has extremely high biological significance. In each element route, highly correlated hub metabolites with |kME| > 0.8 are screened (where the KME value represents the fit between the metabolite and the element route); there are 18 metabolites in total (Figure 5A), and 1–2 are screened in each line based on biological significance and network connectivity. A total of five metabolites were screened as markers of nutritional quality in correlation analysis (Figure S1). Metabolite–nutritional quality and Spearman correlation analyses revealed the differential correlation patterns of the three stoichiometry lines (Figure 5B). The N line hub metabolite, palmitoylcarnitine (lipid, kME = 0.82), showed a significant negative correlation with amino acid content (r = −0.71) and alcohol-soluble content (r = −0.91). The P line hub metabolites showed a positive regulatory pattern: Furfuranol was negatively correlated with triterpenes (r = −0.92) and positively correlated with alkali-soluble polysaccharides (r = 0.73). Methanandamide was positively correlated with alcohol-soluble substances (r = 0.80) and poria cocos acid (r =0.64). The hub metabolites of the N/P line, including Stearamide and Methionylleucine, were negatively correlated with polysaccharide indicators (WPCP, APCP) (r = −0.61 to −0.78).

## 4. Discussion

### 4.1. The N, P, and N/P Ratio Independently Shape the Metabolome

Bagged materials comprise fine materials such as corn kernels and rice bran, which are abundant in cellulose, hemicellulose, crude protein, fat, and other nutrients [9]. These components are more readily degraded and absorbed by fungi, thereby enhancing the production of acetyl-CoA and facilitating the synthesis of triterpenoids [23]. Consequently, the provision of rich nutritional supplementation promotes the accumulation of these compounds. Additionally, pine needles possess higher nitrogen contents than pine woods and are rich in protein and amino acids [24]. They serve as excellent sources of plant protein, offering high nutritional value and aiding in the accumulation of amino acids in *P. cocos*. The difference in tree species and their physical and chemical characteristics affects the quality of *P. cocos*.

The RDA shows that N, P, and N/P diverge in various ways, and the results of the Venn diagram show an extremely small overlap (0%). This indicates that the chemical properties of the substrate influence the overall metabolites of *P. cocos* through independent pathways. Nitrogen and phosphorus, as essential nutrients for the synthesis of proteins, nucleic acids, and lipids, play a critical role in the basal metabolism, growth, and development of fungi [25,26]. Previous studies have demonstrated that nitrogen deficiency restricts the growth of *Ganoderma lucidum* mycelium while promoting the intracellular synthesis of ganoderic acid [27]. Phosphorus deficiency enhanced the synthesis of triacylglycerol in *Rhodosporidium toruloides*, while inhibiting the tricarboxylic acid cycle [28]. *P. cocos*, as a soil microorganism, is related to the cycle information of nitrogen and phosphorus in its cultivation materials [29], and nitrogen and phosphorus have a significant impact on the metabolites of *P. cocos* by affecting metabolic pathways. Lv et al. have studied differentially expressed genes related to fatty acid synthesis in *Aspergillus oryzae* under different conditions with limited N and P elements. The results show that N and P elements control different fatty acid expression genes in Aspergillus oryzae, the flow direction of carbon, and act independently on different metabolite models [30].

### 4.2. N and P Regulate Distinct Metabolic Modules with Opposite Hub Patterns

To identify the specific metabolic modules and metabolites underlying each of these three independent stoichiometric signals, we performed WGCNA to classify metabolites into three “lines” based on their module membership with each factor. Previously, Li et al. used WGCNA to identify metabolites related to AG tolerance in rice seeds [31]; Zhou et al. used the WGCNA metabolite module to identify the triterpene content of Ganoderma lucidum [32]. N (ME1, ME2, ME6, and ME7) and P elements (ME2, ME3, and ME4) respond to different modules: the N/P ratio shares modules ME3 and ME4 with P elements in an exact opposite relationship, indicating that N and P elements influence different metabolites of *P. cocos*. The N/P ratio is balanced in two element lines. To identify the key metabolites of each line, highly correlated hub metabolites with |kME| > 0.8 were screened.

The N line hub (palmitoylcarnitine) is significantly negatively correlated with amino acid and alcohol-soluble contents. Palmitoylcarnitine is a mitochondrial fatty acid oxidation intermediate [33], which is related to intracellular fatty acid metabolism, suggesting that the weakening of fatty acid transport under N-sufficient conditions may be accompanied by amino acid accumulation. The P line hub (Furfuranol and Methanandamide) is negatively correlated with triterpenes and positively correlated with alkali-soluble polysaccharides, alcohol-soluble substances, and poria cocos acid, indicating that the downregulation of triterpene synthesis occurs simultaneously with the accumulation of polysaccharides under P-sufficient conditions. Wang et al. pointed out that under the condition of sufficient P, the glycolysis and pentose phosphate pathways of oleaginous yeast are upregulated, and the carbon flow shifts to polysaccharide accumulation [28]. The hub of the N/P line (Stearamide and Methionylleucine) is negatively correlated with polysaccharide indicators (WPCP and APCP), reflecting the distribution trade-off of the carbon skeleton between lipid amides and polysaccharides under P limitation. Wang et al. found that P limitation activated RNA degradation in oleaginous yeast but inhibited ribosome biogenesis and the TCA cycle, resulting in increased carbon flux in lipids [28]. However, it should be pointed out that the above correlation model is based on correlation analysis of steady-state metabolite abundance, and the final confirmation of carbon flow direction and causal relationship requires stable isotope tracing and targeted metabolomics verification.

### 4.3. Cultivation Implications and Limitations

Although the traditional pine segment cultivation (CK) matrix elements have a balanced ratio (N/P = 1.82), the nitrogen content is extremely low (0.23%), which constitutes the core bottleneck for quality improvement. The N-responsive module ME1 was negatively associated with N availability under this N-deficient condition; the N-line hub metabolite palmitoylcarnitine remains at a high level, suggesting that persistent fatty acid β-oxidation may compete for carbon skeletons that are otherwise available for nitrogenous compound biosynthesis. Consistently, CK exhibited the lowest amino acids (2.59%), poria coco acid (169 mg/kg), and triterpenes (0.20%). However, CK has relatively sufficient phosphorus (0.13%); no P limitation; a normally functioning P pathway ME4; the hub metabolite Furfuranol, which is highly expressed without the initiation of triterpene synthesis; and the polysaccharides WPCP (0.60%) and APCP (83%), which are maintained at high levels. The overall performance of CK is described as a “low nitrogen–low drug efficacy–high polysaccharide” pattern.

The P1 bag material uses pine wilt wood chips as the main matrix, and the element ratio (N/P = 5.63) is in the stoichiometric balance window. The N content (0.60%) was associated with positive ME1 module activity, which had the highest contents of poria coco acid (784 mg/kg), triterpenes (0.82%), and amino acids (5.61%). Simultaneously, the P content (0.11%) also ensures a normal ME4 pathway, and the polysaccharide WPCP (0.62%) is not only undamaged but slightly higher than CK. The statistical conclusion that N and P share less than 5% of the variance in RDA variance decomposition has been biologically verified here: the N pathway was predominantly associated with medicinal ingredients, and the P pathway was linked to the accumulation of polysaccharides. The two pathways operate independently and are not mutually exclusive. This conclusion is consistent with the results of Wang et al.’s study on the volatile components of different cultivated *P. cocos* [9].

The P2 bag material uses oak wood chips as the main matrix, representing a notable case of skewed stoichiometric balance. The N/P ratio is as high as 12.59, and P is extremely deficient (0.05%): the P-responsive module ME4 showed a strong negative association with polysaccharide accumulation; polysaccharides WPCP (0.36%) and APCP (39%) dropped to the lowest levels among the four groups; and alcohol-soluble substances (1.70%) were also severely blocked. N/P line fatty amide hub metabolites (Stearamide, Oleamide, and Linoleamide) are highly expressed under this condition, and all have strong negative correlations with WPCP and APCP (r = −0.61 to −0.78). However, the N content (0.66%) was normal, the N pathway ME1 was unaffected, and triterpene (0.83%) synthesis continued to advance, reaching the highest level among the four groups. These observations suggest that under severe phosphorus limitations, carbon allocation may shift away from carbohydrate polymers toward terpenoid biosynthesis; however, direct carbon flux measurements would be required to confirm this interpretation.

The P3 bag material uses pine needles and pine branch chips as the main matrix. The N content (1.07%, 4.7 times that of CK) and P content (0.16%) are the highest among the four groups, and the N/P ratio (6.64) is within the balance window. The ME1 pathway is highly activated, with amino acids (7.68%) and alcohol-soluble substances (6.76%) reaching the highest levels among the four groups. However, although triterpenes (0.62%) are significantly higher than CK, they are lower than P1 and P2. This observation suggests a potential trade-off: N is sufficient to initiate the synthesis of medicinal ingredients, but when N is excessive, the carbon flow is excessively biased toward amino acids, and triterpene synthesis underperforms compared to P1 with a more balanced N/P ratio. P3 shows that in the quality control of *Poria cocos*, the balance of the N/P ratio is more critical than the absolute content of N.

## 5. Conclusions

A comprehensive evaluation of all indicators found that compared with ordinary wood segment cultivation, the nutritional content of *Poria cocos* was improved. At the same time, the main medicinal ingredients (polysaccharides, terpenes, and poria coco acid) were directly affected by the content and ratio of N and P elements in the matrix. Among them, the P1 pine wilt wood bag material group performed the best. On one hand, it performed excellently for various nutritional indices, with rich contents of poria coco acids and triterpenes, and no significant difference in polysaccharide content, compared with the CK group. On the other hand, the bag material treatment enabled the centralized processing of diseased pine wood and the sustainable development of resources. The P3 pine needles and twigs group performed second-best; it was rich in amino acids and alcohol content, but lacked polysaccharide content. The P2 oak group performed the worst, but identified a tree species that could replace *Pinus massoniana* trees for planting *P. cocos* in the future. The specific effects of the matrix on the nutritional content of *P. cocos* are as follows: RDA and variance decomposition showed that N, P, and the N/P ratio independently regulate various metabolic modules (the shared part is less than 5%). WGCNA further revealed the correlation pattern between third-line metabolites and nutritional quality: the N response module is positively correlated with the accumulation of triterpenes and amino acids, but its hub metabolites are negatively correlated. As such, N may indirectly promote the accumulation of nitrogen-containing nutrients by inhibiting the release of carbon skeletons from lipid metabolism. The P response module is highly correlated with polysaccharides (WPCP/APCP), showing a positive regulatory mode; the N/P ratio module is shared with the P line but in the opposite direction, indicating that polysaccharide synthesis is mainly limited by P availability. Hub metabolite–nutrition correlation analysis revealed a consistent pattern suggestive of stoichiometric resource allocation: lipid amide accumulation is accompanied by a decrease in polysaccharide synthesis under P-limiting conditions, carbon flow occurs in the direction of polysaccharides, and alcohol-soluble products are affected by P-sufficient conditions. This model needs further experimental verification.

These findings provide a quantitative basis for optimizing substrate formulations, making it possible to directionally modulate target metabolites without compromising biological efficiency by adjusting the nitrogen-to-phosphorus ratio. Targeted metabolomics could be employed to explore multi-component synergistic effects in the future. In addition, the molecular mechanisms underlying substrate-driven metabolic regulation—such as differences in the expression of key enzyme genes—require validation through functional studies such as gene knockout and overexpression experiments. Such efforts could elucidate the regulatory pathways of nitrogen and phosphorus in amino acid and fatty acid synthesis, providing molecular-level evidence of the precision formulation of bag substrates.

## Figures and Tables

**Table 1:** Nutritional composition of *P. cocos*.

Nutrition	CK	P1	P2	P3
Moisture (%)	7.25 ± 0.07 ^b^	7.51 ± 0.05 ^a^	7.63 ± 0.03 ^a^	7.60 ± 0.03 ^a^
Ash content (%)	0.0357 ± 0.0006 ^c^	0.0420 ± 0.0011 ^b^	0.0453 ± 0.0004 ^a^	0.0404 ± 0.0007 ^b^
Alcohol-soluble substances (%)	4.00 ± 0.01 ^c^	5.66 ± 0.08 ^b^	1.70 ± 0.02 ^d^	6.76 ± 0.02 ^a^
Total triterpenoids (%)	0.2036 ± 0.0056 ^c^	0.8162 ± 0.0184 ^a^	0.8284 ± 0.0086 ^a^	0.6155 ± 0.0042 ^b^
poria cocos acid (mg/kg)	168.95 ± 3.02 ^d^	783.94 ± 3.39 ^a^	413.34 ± 2.80 ^c^	611.63 ± 1.63 ^b^
WPCP (%)	0.5985 ± 0.0015 ^b^	0.6242 ± 0.0059 ^a^	0.3568 ± 0.0031 ^d^	0.5888 ± 0.0015 ^c^
APCP (%)	83.05 ± 0.16 ^a^	71.89 ± 0.19 ^b^	39.37 ± 0.55 ^d^	56.73 ± 0.30 ^c^
Total amino acids (%)	2.59 ± 0.01 ^d^	5.61 ± 0.02 ^c^	5.51 ± 0.03 ^b^	7.68 ± 0.07 ^a^
biological conversation efficiency (%)	12.80 ± 0.64 ^c^	31.72 ± 1.47 ^a^	11.32 ± 1.03 ^d^	28.68 ± 1.02 ^b^

Note: Different letters in the same row indicate statistically significant differences (*p* < 0.05).

## Data Availability

Data will be made available on request.

## Supplementary Materials

The following supporting information can be downloaded at https://www.mdpi.com/article/10.3390/jof12090637/s1. Figure S1: 5 different metabolites; Table S1: The biological conversation efficiency.
