# Supplementary material for: Differences in Nutritional Composition of Poria cocos Cultivated with Different Raw Materials Based on Non-Targeted Metabolomics Method
Source: J Fungi (Basel). 2026 Aug 26;12(9):637. doi: 10.3390/jof12090637 (PMC13608800; doi:10.3390/jof12090637)
Supplement: Supplementary file 1 [file jof-12-00637-s001.zip › jof-4460344-supplementary.pdf]

Supporting Information

Differences in nutritional composition of *Poria cocos* cultivated with different raw materials based on non-targeted metabolomics method

Yusong Li[a,b], Jianbin Xu[c], Chunlai Yu[c,*], Jinping Zhang[a,*], Yinan Wang[a], Zeyu Zhang[a], Fengqing Li[d], Kaitai Yang[e]

[a] Subtropical Forestry Research Institute, Chinese Academy of Forestry, Hangzhou, 311400, China

[b] College of Landscape Architecture, Nanjing Forestry University, Nanjing, 210037, China

c Chunan County Forestry Bureau, Hangzhou, 311700, China

[d] Experimental center of subtropical Forestry, Chinese Academy of Forestry, Xinyu, 336603, China

[e] Guanxi Zhuang Autonomous Region Academy of Forestry, Nanning, 530002, China

[*] Correspondence to: Yu: casfz@163.com; Zhang: jinpingzhang@126.com

Supporting Information description

Figure S1. 5 different metabolites.

Table S1. The biological conversation efficiency.

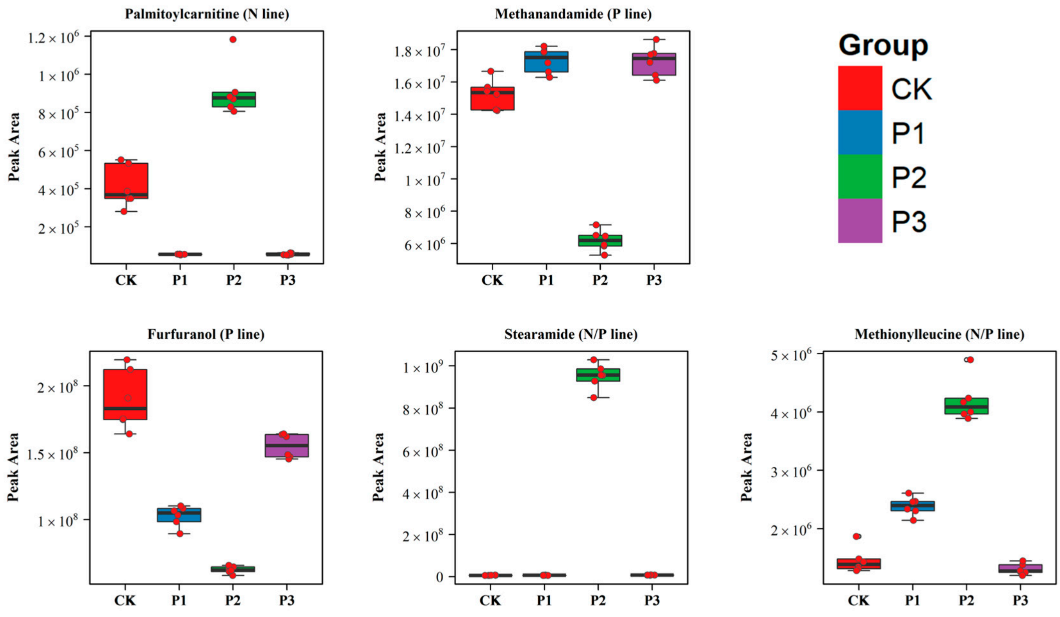

Figure S1. 5 different metabolites: N line Hub metabolite: Palmitoylcarnitine; P line Hub metabolite: Furfuranol and Methanandamide; N/P line metabolite: Stearamide and Methionylleucine.

Table S1. The biological conversation efficiency.

Group	Raw material(kg)	*P.cocos*(kg)	ratio
CK	6.04±0.25	0.77±0.06	0.13±0.01
P1	6.11±0.19	1.92±0.15	0.32±0.01
P2	6.09±0.18	0.68±0.05	0.11±0.01
P3	5.92±0.20	1.70±0.11	0.29±0.01

Note: each group had cutivated with 30 cellars, those date cames from 5 cellars in each group radomlly.
